# Adhesive Properties of Aromatic Polyesters with Cationic Side Chains Synthesized via Radical Ring-Opening Copolymerization

**DOI:** 10.1021/acsomega.6c06129

**Published:** 2026-08-02

**Authors:** Gaku Sugimoto, Yoshiaki Kobayashi, Syuuhei Komatsu, Shohei Shiomoto, Akihiko Kikuchi, Taka-Aki Asoh

**Affiliations:** † Department of Materials Science and Technology, 26413 Tokyo University of Science, Tokyo, Japan 125-8585; ‡ Faculty of Pharmacy and Pharmaceutical Sciences, Josai University, Sakado, Japan 350-0295; § Department of Medical and Robotic Engineering Design, 26413 Tokyo University of Science, Tokyo, Japan 125-8585

## Abstract

Degradable adhesives that are both strong and cleanly debondable are highly desirable for progressing sustainable materials but are challenging to develop owing to the limited availability of polymer systems that are endowed with both sufficient cohesion and interfacial interactions. Herein, we report a series of novel P­(METATFSI-*co*-BMDO) polymers as a new class of cationic-side-chain-bearing aromatic polyester synthesized via radical ring-opening copolymerization of [2-(methacryloyloxy)­ethyl]­trimethyl­ammonium bis­(trifluoro­methanesulfonyl)­imide (METATFSI), an ionic-liquid monomer, and 5,6-benzo-2-bromomethyl-1,3-dioxepane (BMDO), an aromatic cyclic ketene acetal. Incorporating BMDO endows the polymer backbone with ester linkages that facilitate main-chain degradability, while the combination of its aromatic units and dense cationic side chains provides pronounced cation−π interactions. The use of the ionic-liquid monomer significantly enhances the BMDO-polymerization efficiency to afford copolymers in high yields and with tunable compositions. Strong cation−π interactions are evidenced by lower solubilities and higher glass transition temperatures compared to those predicted by ideal mixing, indicative of superior cohesive interactions within the polymer matrix. Adhesion testing revealed that P­(METATFSI-*co*-BMDO) is strongly adhesive toward aluminum as well as challenging low-surface-energy substrates, including polypropylene and polytetrafluoroethylene. Intermediate copolymer compositions led to optimal adhesion, which reflects a balance between cohesive strengthening via cation−π interactions and effective interfacial adhesion through ionic and dipolar interactions. Hence, radical ring-opening copolymerization is a powerful strategy for integrating cation−π interactions into degradable polyester adhesives, thereby offering a promising platform for high-performance, sustainable adhesive materials that are applicable to a wide range of substrates.

## Graphical abstract

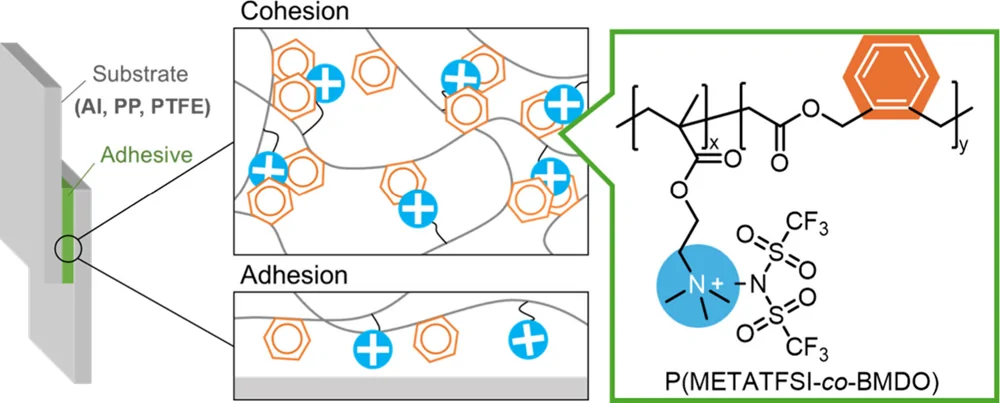

## Introduction

Adhesives play essential roles in numerous industrial and consumer applications, including packaging, construction, electronics, automotive engineering, and biomedical devices. Their performance is governed by a balance between cohesion, originating from intermolecular interactions within the adhesive, and interfacial adhesion, derived from interactions between the adhesive and substrate.^1,2^ However, conventional strong adhesives often degrade poorly and are not easily removed from substrates, which prevents material recycling and causes to environmental waste.^3,4^ Therefore, the development of degradable adhesives that are both strongly adhesive and cleanly detachable has emerged as an important challenge in the sustainable materials science field.^5^

Polyesters are attractive building blocks for degradable polymeric materials because main-chain ester linkages can be hydrolytically cleaved under mild conditions.^6^ While commercial polyesters such as poly­(lactic acid) (PLA), poly­(caprolactone) (PCL), and poly­(butylene succinate) (PBS) are biodegradable, they generally lack functional groups that provide strong cohesive or interfacial interactions when used as adhesives. Moreover, their simple ring-opening-polymerization-derived backbone structures intrinsically hinder the direct introduction of reactive side-chain functionalities. Although copolymerization with functionalized lactones, such as α-substituted ε-caprolactones, has been reported to be a viable strategy for imparting side-chain functionality,^7^ incorporating adhesion-promoting groups into a biodegradable polyester framework remains challenging using conventional synthetic approaches.

Cation−π interactions, which occur between cationic groups and aromatic π-electron systems, have recently gained attention as noncovalent forces capable of significantly enhancing both cohesion and interfacial adhesion in a synthetic polymer.^8–12^ These interactions have been widely applied to waterborne and bioinspired adhesives in which their ability to strengthen bonding even under challenging conditions has been demonstrated. Fundamental studies have revealed that cation−π interactions can generate substantial binding forces in aqueous environments and contribute significantly to the adhesion mechanisms of mussel-inspired proteins.^13,14^ Despite these advantages, strong cation−π interactions have not yet been effectively incorporated into degradable polyester systems, which is mainly ascribable to monomer-availability limitations and incompatibility between the units that underdoing ring-opening polymerization and the cationic functionalities. Recent studies have demonstrated that polyester adhesive performance can be effectively improved through oxygen-rich structures and crystallinity engineering.^15,16^ However, the use of cation−π interactions to simultaneously enhance cohesion and adhesion in degradable polyester adhesives remains largely unexplored.

The radical ring-opening copolymerization of a cyclic ketene acetal (CKA), such as 2-methylene-1,3-dioxepane (MDO), and 5,6-benzo-2-methylene-1,3-dioxepane (BMDO) affords a polymer with ester bonds in its main chain, thereby facilitating degradability while maintaining vinyl copolymerization versatility.^17^ Developing biodegradable materials by copolymerizing vinyl monomers with CKAs has been studied for decades and remains an actively investigated research field. We previously reported the synthesis of degradable polymeric microparticles and thermoresponsive polymers by copolymerizing hydrophobic MDO with hydrophilic vinyl monomers.^18–20^ Kohsaka et al. reported that copolymers containing BMDO undergo fast and selective main-chain scission via domino reactions triggered by pendant-group activation, which facilitates rapid molecular-weight loss under mild conditions.^21^

BMDO is particularly attractive because it introduces aromatic moieties, potential partners for cation−π interactions, into the polymer backbone. Meanwhile, the cationic monomer provides a densely cationic methacrylate unit capable of strongly binding through electrostatic and cation−π interactions. However, CKAs propagate slowly during radical polymerization; hence, precisely controlling the copolymer composition is difficult during copolymerizations involving comonomers.^17,22^ Consequently, bulk polymerization is often preferred over solution polymerization for CKA-based systems. In contrast, radical polymerization reportedly propagates faster in an ionic liquid,^23,24^ and Ueki et al. reported a radical-polymerization-based method for synthesizing high-molecular-weight polymer gels in ionic liquids by exploiting this feature.^25,26^

With the above-mentioned considerations in mind, we hypothesized that the radical ring-opening copolymerization of [2-methacryloyloxy)­ethyl]­trimethyl­ammonium bis­(trifluoro­methanesulfonyl)­imide (METATFSI), an ionic-liquid monomer, with BMDO, and aromatic CKA, is a versatile strategy for constructing degradable adhesive polymers endowed with main-chain degradability and strong noncovalent interactions. In particular, incorporating aromatic units into the polyester backbone along with densely cationic side chains is expected to lead to pronounced intra- and intermolecular cation−π interactions that are anticipated to play a dual role by enhancing the cohesive strength of the adhesive matrix while simultaneously promoting interfacial adhesion to both high- and low-surface-energy substrates. Effectively integrating cation−π interactions into degradable polyester adhesives as well as their quantitative contributions to cohesion and interfacial adhesion remains poorly understood, despite having been extensively studied in hydrogels and nondegradable polymer systems. Therefore, we not only aimed to synthesize a series of aromatic P­(METATFSI-*co*-BMDO) polyesters bearing cationic side chains via radical ring-opening copolymerization, but also to systematically elucidate how cation−π interactions govern their physicochemical properties and adhesive performance (Figure 1). Specifically, we investigated how the copolymer composition affects the solubility, thermal behavior, and adhesive performance of the product.

**Figure 1:**
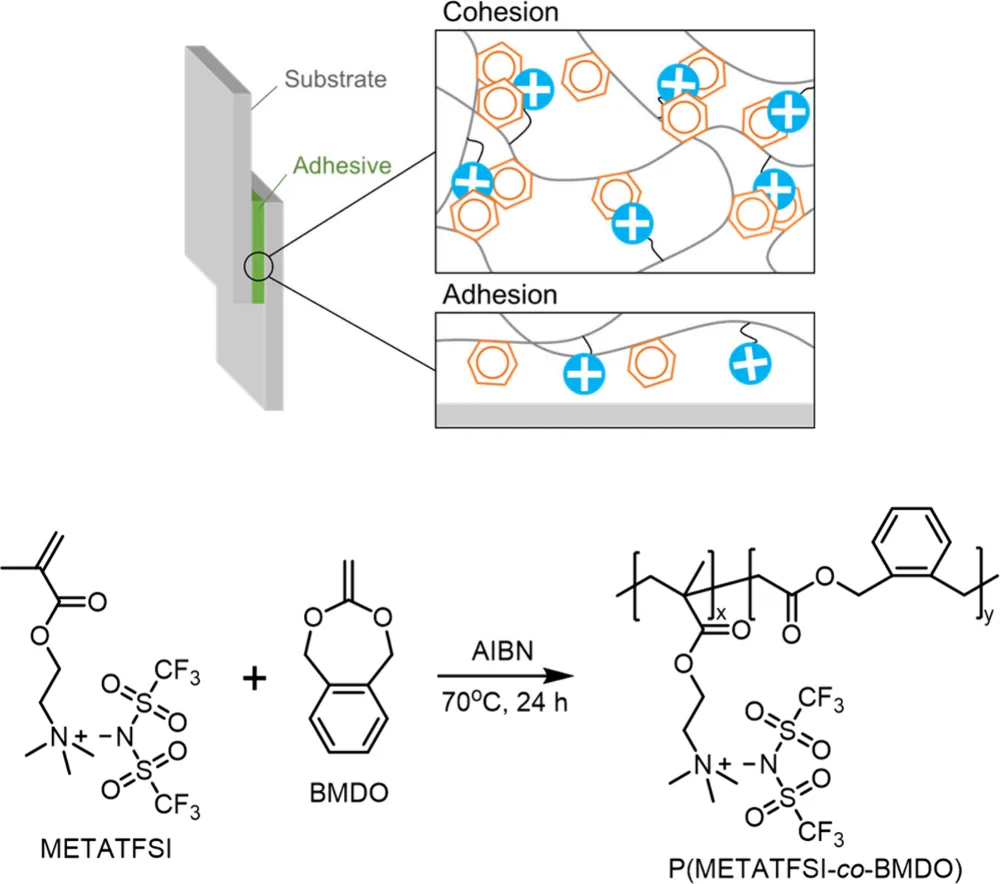
Schematic depicting the synthesis of aromatic polyesters bearing cationic side chains (P­(METATFSI-*co*-BMDO)) via the radical ring-opening copolymerization of the METATFSI ionic-liquid monomer and the BMDO aromatic cyclic ketene acetal. Ester linkages are introduced into the main chain through the ring opening of BMDO, while cationic side chains are incorporated via METATFSI.

## Experimental Section

### Materials

1,2-Benzenedimethanol, bromoacetaldehyde diethyl acetal, *p*-toluenesulfonic acid monohydrate, and lithium bis­(trifluoro­methanesulfonyl)­imide (LiTFSI) were purchased from TCI (Japan). Potassium *tert*-butoxide (*t*-BuOK), *tert*-butanol (*t*-BuOH), and 2,2′-azobis­(isobutyronitrile) (AIBN) were purchased from Wako Pure Chemical Industries (Japan). In addition, [2-(methacryloyloxy)­ethyl]­trimethyl­ammonium chloride 75 wt % in H_2_O (METACl) and *N*,*N*-dimethylaminoethyl methacrylate (DMAEMA) were purchased from Sigma–Aldrich (USA). BMDO was prepared by the acid-catalyzed acetalization of 1,2-benzenedimethanol and bromoacetaldehyde diethyl acetal followed by subsequent dehydrohalogenation of the formed 5,6-benzo-2-bromomethyl-1,3-dioxepane with *t*-BuOK in *t*-BuOH.^27,28^ METATFSI was synthesized via ion-exchange involving METACl and LiTFSI as previously reported.^29^ Briefly, LiTFSI was dissolved in water and added to a vigorously stirred aqueous solution of METACl, which led to the formation of two liquid phases. The mixture was stirred at elevated temperature, after which the upper aqueous phase was removed by decantation. The remaining ionic liquid was washed with water to remove unreacted species and inorganic salts, and then dried by evaporation to afford METATFSI, whose structure was confirmed by ^1^H nuclear magnetic resonance (NMR) spectroscopy, which revealed the presence of signals expected for its methylene and aromatic protons.

### BMDO/Vinyl Monomer Radical Copolymerization

BMDO and a vinyl monomer (METATFSI or DMAEMA) were subjected to radical copolymerization in the bulk under nitrogen using 1 mol % AIBN as the initiator. To the end, BMDO and AIBN were dissolved in the corresponding comonomer; polymerization was conducted at 70 °C for 24 h with comonomer/BMDO feed ratios that ranged between 0:100 and 100:0. The resulting crude polymers were purified by repeated precipitation from hexane, with subsequent centrifugation (four cycles) and drying under vacuum.

### Characterization

The chemical structures of the various polymers were determined by ^1^H NMR spectroscopy. Each sample (∼5 mg) was dissolved in an appropriate deuterated solvent (1 mL). The resulting solution was filtered through absorbent cotton and transferred to an NMR tube. The obtained spectra were analyzed using 1D NMR software, with the polymer structures determined from the ratios of the integrated signals. The thermal properties of the various P­(METATFSI-*co*-BMDO) samples were evaluated by differential scanning calorimetry (DSC). A sample (6.6 mg) was sealed in an aluminum pan and heated from – 30 to 200 °C at 10 °C min^–1^ in air, with first and second heating runs recorded. The glass transition temperature (*T*_g_) was determined from the second heating scan. Static water contact angles of Al, PP, and PTFE substrates were measured at room temperature.

### Adhesion Testing

Prior to adhesion testing, Al, PP, and PTFE substrates were washed with acetone and dried under ambient conditions. The cleaned substrates were used without further surface treatment. The water contact angles of the cleaned Al, PP, and PTFE substrates were 80 ± 1°, 87 ± 2°, and 105 ± 1°, respectively. Adhesive formulations were prepared by dissolving or dispersing each polymer in acetone to a concentration of 5, 10, 15, or 20 wt %. A 20-μL aliquot of each adhesive was applied to the edge of a 10 × 25 mm aluminum (Al) substrate. A second substrate was then placed on top of the first to create a 10 × 10 mm bonding area, and the assembly was fixed using an all-plastic pinch clip. The substrates were bonded at 25 °C, with samples allowed to dry for 24 h (or 1–2 weeks in the case of P­(DMAEMA-*co*-BMDO)). Lap shear testing was then used to measure adhesive strengths. A 14.4-μL adhesive sample was used when DMSO was employed as the solvent or dispersant, with bonding carried out at 60 °C and drying performed in a constant-temperature oven for 3–4 days. The specimens were subsequently cooled at 25 °C for 10 min prior to shear testing. Adhesion testing was performed in triplicate under each set of conditions, with average adhesive strengths calculated.

## Results and Discussion

The feed ratios, copolymer compositions, and yields of the synthesized P­(METATFSI-*co*-BMDO) and P­(DMAEMA-*co*-BMDO) samples are summarized in Table 1, with Figure 2a showing how the BMDO feed ratio affects the BMDO content in the P­(METATFSI-*co*-BMDO) copolymer and its yield. The corresponding results for P­(DMAEMA-*co*-BMDO) samples prepared under identical polymerization conditions are shown in Figure 2b for comparison. P­(METATFSI-*co*-BMDO), which was prepared by copolymerizing BMDO with the METATFSI ionic liquid monomer, exhibited a BMDO content that increased with increasing BMDO feed ratio, which reveals that the copolymer composition can be controlled relatively well. Moreover, with the exception of the PBMDO homopolymer, high yields (∼80–90%) were obtained for all systems. In contrast, the BMDO content in the resulting copolymer was significantly lower than the corresponding BMDO feed ratio when BMDO was copolymerized with the DMAEMA nonionic-liquid monomer, with copolymer yield observed to decrease with increasing BMDO content. These results are consistent with previous studies involving nonionic-liquid monomers, which validates our current observations.^30,31^ The ring-opening radical polymerization of a cyclic ketene acetal, such as BMDO, is well-known to proceed very slowly, and copolymerization with comonomers often results in low yields and copolymer compositions that are poorly controlled.^17–20,22,30^ On the other hand, vinyl monomers capable of undergoing radical polymerization reportedly exhibit higher polymerization rates in ionic liquids.^23–26^ Therefore, the METATFSI ionic-liquid monomer is expected to enhance the polymerization rate of BMDO in the bulk, leading to the quantitative formation of the corresponding copolymer. Although only METATFSI was investigated in this study, other ionic-liquid monomers may influence the copolymerization behavior and adhesive performance. Future studies will explore the effects of ionic-liquid monomer structure, including variations in both cation and anion structures.

**Table 1:** P­(METATFSI-*co*-BMDO) and P­(DMAEMA-*co*-BMDO) Samples Prepared by Radical Ring-Opening Copolymerization^a^

Feed ratio (molar ratio)			Copolymer composition			
METATFSI	DMAEMA	BMDO	METATFSI	DMAEMA	BMDO	Yield (%)
0	-	100	0	-	100	13
10	-	90	29	-	71	78
25	-	75	43	-	57	81
50	-	50	66	-	34	81
75	-	25	79	-	21	95
100	-	0	100	-	0	94
-	10	90	-	48	52	24
-	25	75	-	67	33	37
-	50	50	-	79	21	45
-	75	25	-	93	7	55
-	100	0	-	100	0	98

a Copolymerizations were carried out at 70 °C for 24 h using 1 mol % AIBN (relative to the total monomer feed) as initiator.

**Figure 2:**
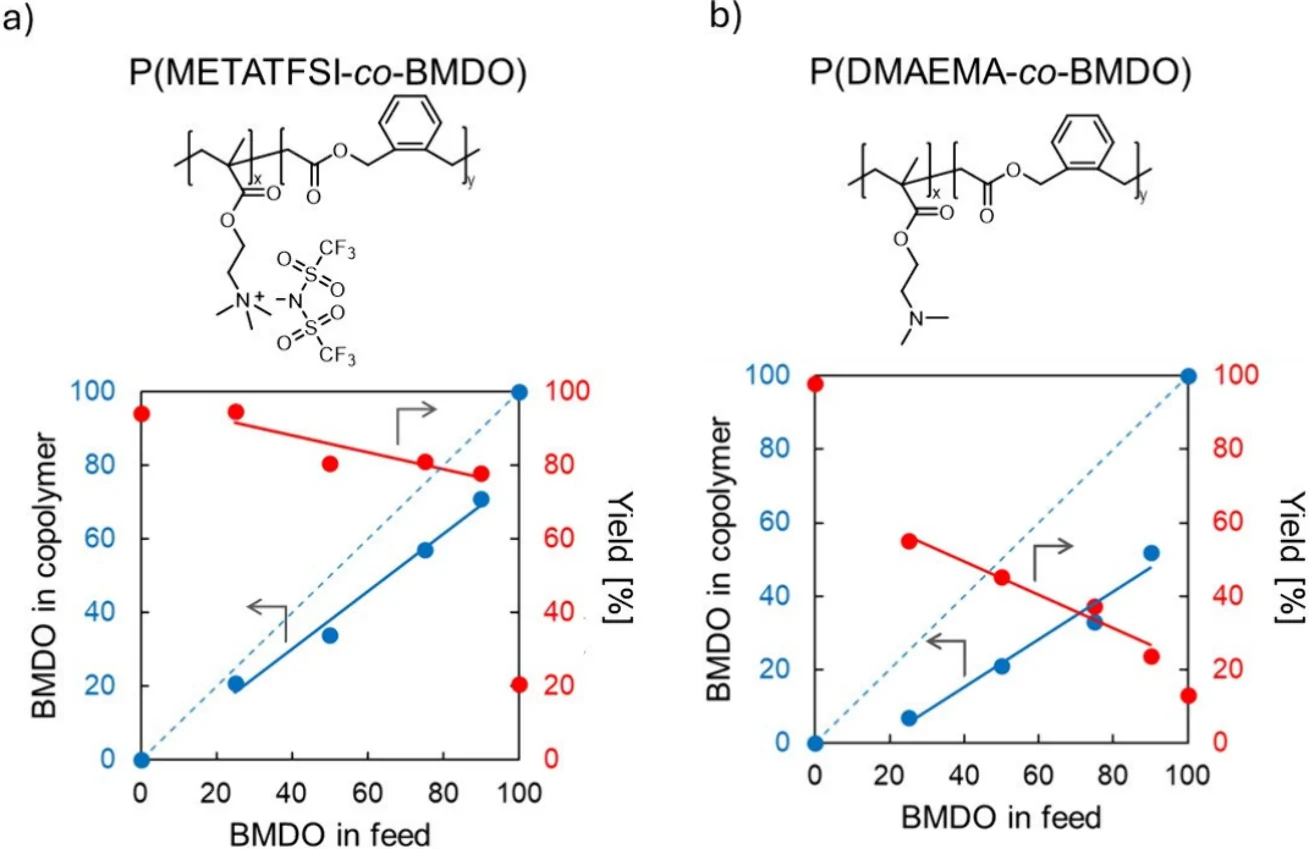
BMDO-feed-ratio dependences of the BMDO content in the copolymer and yield: (a) P­(METATFSI-*co*-BMDO) and (b) P­(DMAEMA-*co*-BMDO).

We evaluated the solubilities of the various P­(METATFSI-*co*-BMDO) samples in DMSO by adding 5 mg of each polymer to 1 mL of DMSO, with photographic images of the various mixtures shown in Figure 3a. Both the PMETATFSI and PBMDO homopolymers are soluble in DMSO. In contrast, the copolymers exhibited solubilities that decreased with increasing BMDO content. Specifically, the copolymer with a 43:57 METATFSI/BMDO composition ratio exhibited slight turbidity, whereas that with the 29:71 ratio was apparently turbid. We conclude that both the METATFSI and BMDO units themselves are soluble in DMSO because both homopolymers are soluble in this solvent. Hence, the reduced solubility is consistent with the presence of intra- and intermolecular cation−π interactions between aromatic rings and cationic moieties within the copolymer. However, other intermolecular interactions, including ionic aggregation and dipole–dipole interactions, may also contribute to the observed aggregation behavior.

**Figure 3:**
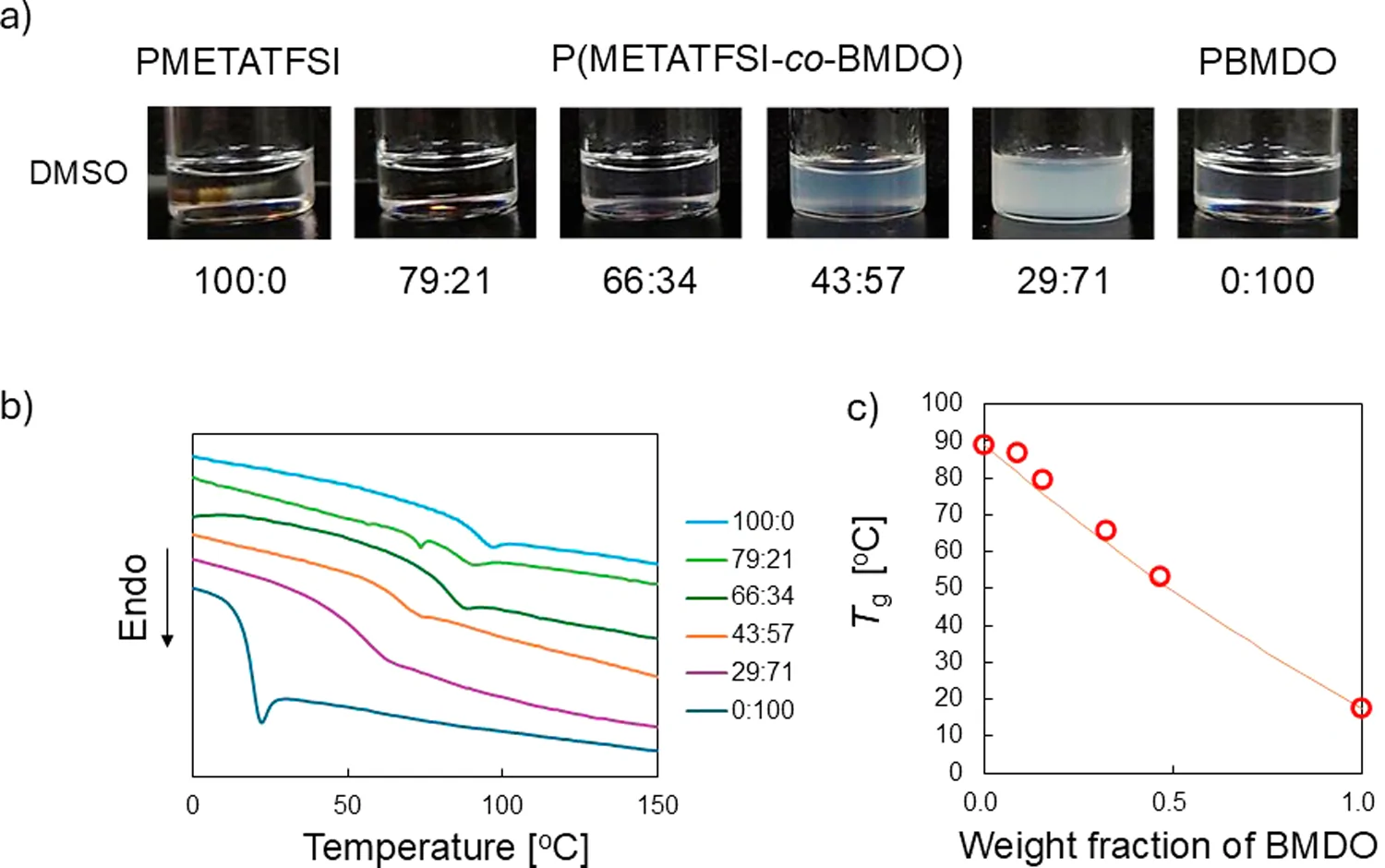
(a) Photographic images showing the solubilities of P­(METATFSI-*co*-BMDO) samples with different METATFSI/BMDO composition ratios in DMSO (5 mg/mL). (b) DSC thermograms of METATFSI/BMDO copolymer composition. (c) *T*_g_ as a function of the METATFSI weight fraction (circles), together with the values predicted by the Fox equation (line).

We used DSC to evaluate the thermal properties of the various P­(METATFSI-*co*-BMDO) samples; the acquired thermograms are displayed in Figure 3b, while the DSC- determined glass transition temperatures (*T*_g,exp_) are summarized in Table 2. Figure 3b reveals that all P­(METATFSI-*co*-BMDO) samples exhibited glass transitions with no melting temperatures (*T*_m_). In addition, P­(METATFSI-*co*-BMDO) exhibited a *T*_g,exp_ that decreased with increasing BMDO content.

**Table 2:** Copolymer Compositions (Molar Ratios and Weight Fractions) and Glass Transition Temperatures of P­(METATFSI-*co*-BMDO) Samples

Copolymer composition (molar ratio)		Copolymer composition (weight fraction)		*T*_g_ (°C)	
METATFSI	BMDO	METATFSI	BMDO	*T*_g_, _exp_ ^a^	*T*_g_,_calc_ (FOX)^b^
0	100	0	1	17.5	17.5
29	71	0.53	0.47	53.1	51.7
43	57	0.68	0.32	65.7	62.5
66	34	0.84	0.16	79.5	75.7
79	21	0.91	0.09	86.9	81.5
100	0	1	0	89.1	89.1

a *T*_g_,_exp_ is the DSC-determined glass transition temperature.

b *T*_g_,_calc_ (FOX) is the glass transition temperature calculated using the Fox equation.

The Fox equation^31,32^ is an ideal mixing rule that is widely used to predict the *T*_g_ value of a random copolymer or polymer blend in the absence of specific interactions, and is given by

$$1/T_{g}=w_{1}/T_{g,1}+w_{2}/T_{g,2}$$

where *w*_1_ and *w*_2_ are the weight fractions of the METATFSI and BMDO units in the copolymer, respectively, and *T*_g,1_ and *T*_g,2_ are the glass transition temperatures of the corresponding PMETATFSI and PBMDO homopolymers. The glass transition temperatures theoretically calculated using this equation (*T*_g,calc_ (Fox)) are also summarized in Table 2. *T*_g,exp_ and *T*_g,calc_ (Fox) values are plotted as circles and a solid line, respectively, in Figure 3c. The experimentally determined *T*_g_ values are slightly higher than those predicted using the Fox equation, which is ascribable to strong intermolecular interactions, such as hydrogen bonds, ionic interactions, or π-mediated interactions that suppress segmental motion, resulting in experimentally recorded *T*_g_ values that exceed those predicted by the Fox equation.^32,33^ Taken together, the solubility and *T*_g_ data are consistent with the presence of cation−π interactions within the copolymer system. However, because direct spectroscopic evidence was not obtained, contributions from other intermolecular interactions, such as ionic aggregation and dipole–dipole interactions, cannot be excluded.

The adhesive strengths of the PMETATFSI, PBMDO, and P­(METATFSI-*co*-BMDO) samples when bonded to aluminum (Al) plates were evaluated using polymer solutions or dispersions in acetone, the results of which are shown in Figure 4. Although the polymers became increasingly less soluble in acetone with increasing BMDO content (Figure 4a), acetone was initially selected as the solvent because it is volatile and evaporates easily during adhesion testing. All adhesion experiments were conducted at 25 °C (Figure 4b). The same adhesion area (10 × 10 mm^2^) and clamping procedure were used throughout the study to ensure consistent testing conditions and enable reliable comparison among the samples. Figure 4c reveals that P­(METATFSI-*co*-BMDO) exhibited a maximum adhesive strength of ∼ 1.7 MPa at a METATFSI/BMDO copolymerization ratio of 66:34, with lower values recorded at other composition ratios. This behavior is attributable to the superior cohesive strength of the adhesive resulting from cation−π interactions within the polymer matrix. In addition, adhesive strength was observed to increase significantly as the polymer concentration increased from 5 to 20 wt %, which indicates that failure occurs predominantly via cohesive rather than interfacial fracturing according to Figure 4c. Therefore, we conclude that cation−π interactions enhance the cohesive strength, which increases the adhesive strength of the adhesive as the METATFSI/BMDO ratio approaches the appropriate value at a sufficiently high polymer concentration.

**Figure 4:**
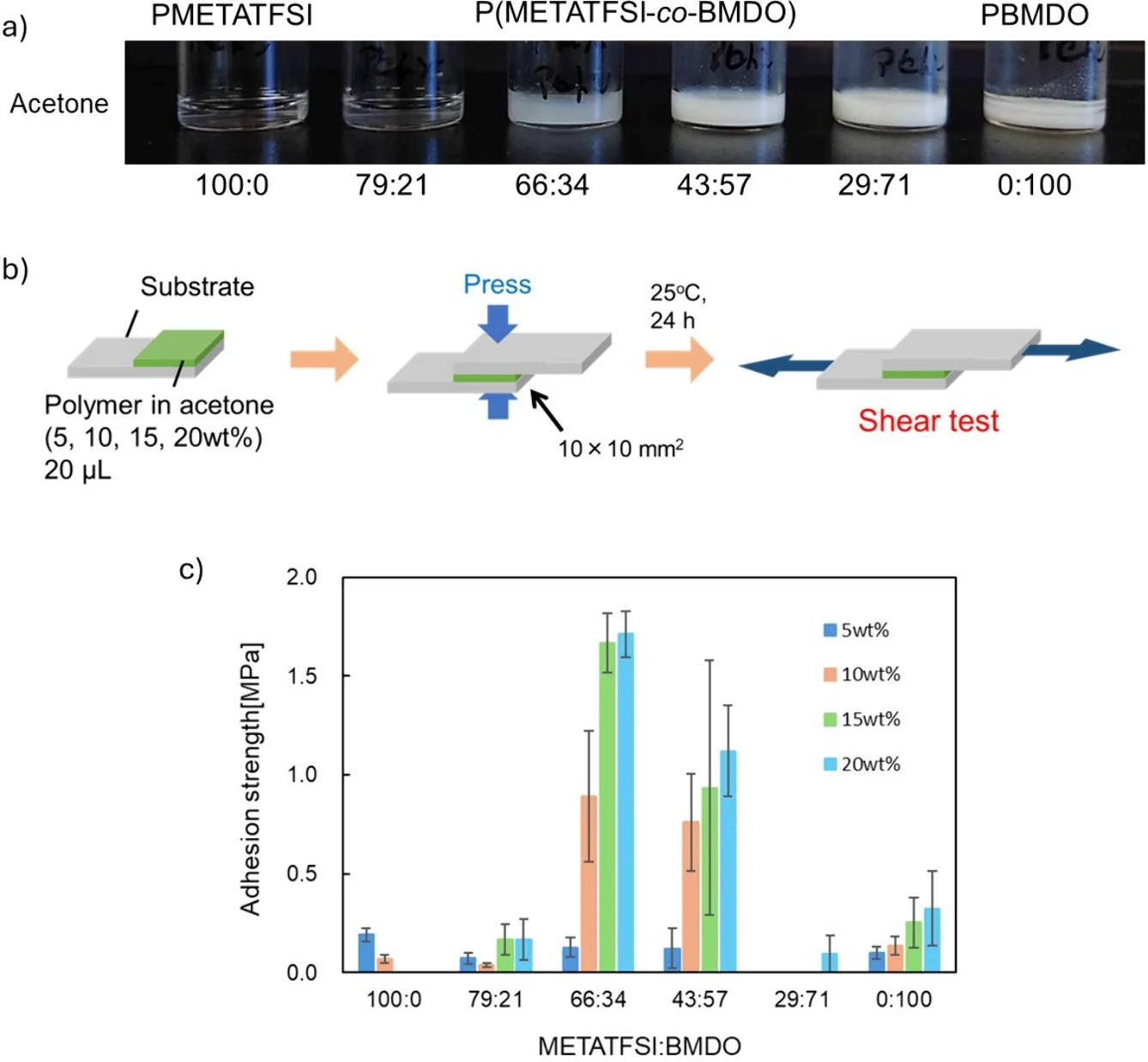
(a) Photographic images showing the solubilities of P­(METATFSI-*co*-BMDO) samples with different METATFSI/BMDO composition ratios in acetone (5 mg/mL). (b) Schematic depicting the adhesion test used to evaluate adhesive strength. (c) Dependence of adhesive strength on the METATFSI/BMDO copolymer composition for adhesive concentrations of 5, 10, 15, and 20 wt % on Al plates, which highlights the composition-dependent adhesion behavior.

On the other hand, while both intra- and intermolecular cation−π interactions are expected to strengthen with increasing BMDO content according to the solubility results provided above, Figure 4c reveals that the copolymer with a METATFSI/BMDO composition ratio of 66:34 exhibited the highest adhesive strength, whereas the 29:71 copolymer exhibited a relatively low value. This discrepancy is attributable to the fact that the P­(METATFSI-*co*-BMDO) sample with the high BMDO content did not actually dissolve well in the solvent but was merely dispersed in acetone during its preparation.

To clarify this point, we also evaluated adhesive strengths using DMSO as the solvent; here, adhesion experiments were conducted at 60 °C to ensure complete evaporation of the DMSO during the bonding process. The drying conditions (60 °C, 3–4 days) were selected because the measured adhesive strength reached an approximately constant value under these conditions. Although residual DMSO is expected to be substantially reduced after prolonged drying, its complete removal was not directly verified. Therefore, a possible contribution of residual DMSO to polymer plasticization and adhesive performance cannot be entirely excluded. All polymer samples exhibited higher adhesive strengths when DMSO was used as the solvent rather than acetone (Figure 4c vs **5a**). The markedly higher adhesive strength exhibited by the PMETATFSI homopolymer and the copolymer with a 79:21 METATFSI/BMDO composition ratio is attributable to the high boiling point of DMSO, which evaporates slowly and enables these highly viscous adhesives sufficient time to wet and adhere to the substrate prior to drying, thereby affording superior interfacial adhesion. Furthermore, the higher adhesive strengths observed for copolymers with METATFSI/BMDO composition ratios of 66:34, 43:57, and 29:71, as well as the PBMDO homopolymer, are attributable to the high BMDO-unit solubility in DMSO. In addition, the elevated drying temperature of 60 °C used for the copolymer with a 29:71 METATFSI/BMDO composition ratio and the PBMDO homopolymer, which exceeds their *T*_g_ values, is also expected to contribute to their superior adhesive strengths.

The ester linkages in the main chain of the copolymer endow it with enhanced cohesive strength arising from intra- and intermolecular cation−π interactions, as well as main-chain cleavability, which is advantageous, as discussed above. We confirmed the formation of soluble aromatic degradation products in preliminary alkaline hydrolysis experiments (Figure S1), supporting the hydrolytic cleavability of the ester-containing backbone. However, a certain PBMDO + PMETATFSI homopolymer mixture should also deliver maximum adhesive strength considering the adhesive strengths that arise solely through intermolecular interactions. Therefore, we tested adhesion using PMETATFSI + PBMDO homopolymer mixtures with 75:25, 50:50, and 25:75 molar ratios, the results of which are displayed in Figure 5b. As expected, the 50:50 homopolymer mixture exhibited the highest adhesive strength; this ratio is close to the optimal 66:34 value for the components in the P­(METATFSI-*co*-BMDO) copolymer. Adhesive strength also decreased as the composition deviated from the optimal ratio. These results suggest that the coexistence of aromatic and cationic components contributes to enhanced adhesive performance in the mixed-polymer system. Although intermolecular cation−π interactions are likely involved, additional factors such as blend morphology and wetting behavior may also influence the observed adhesion strengths.

**Figure 5:**
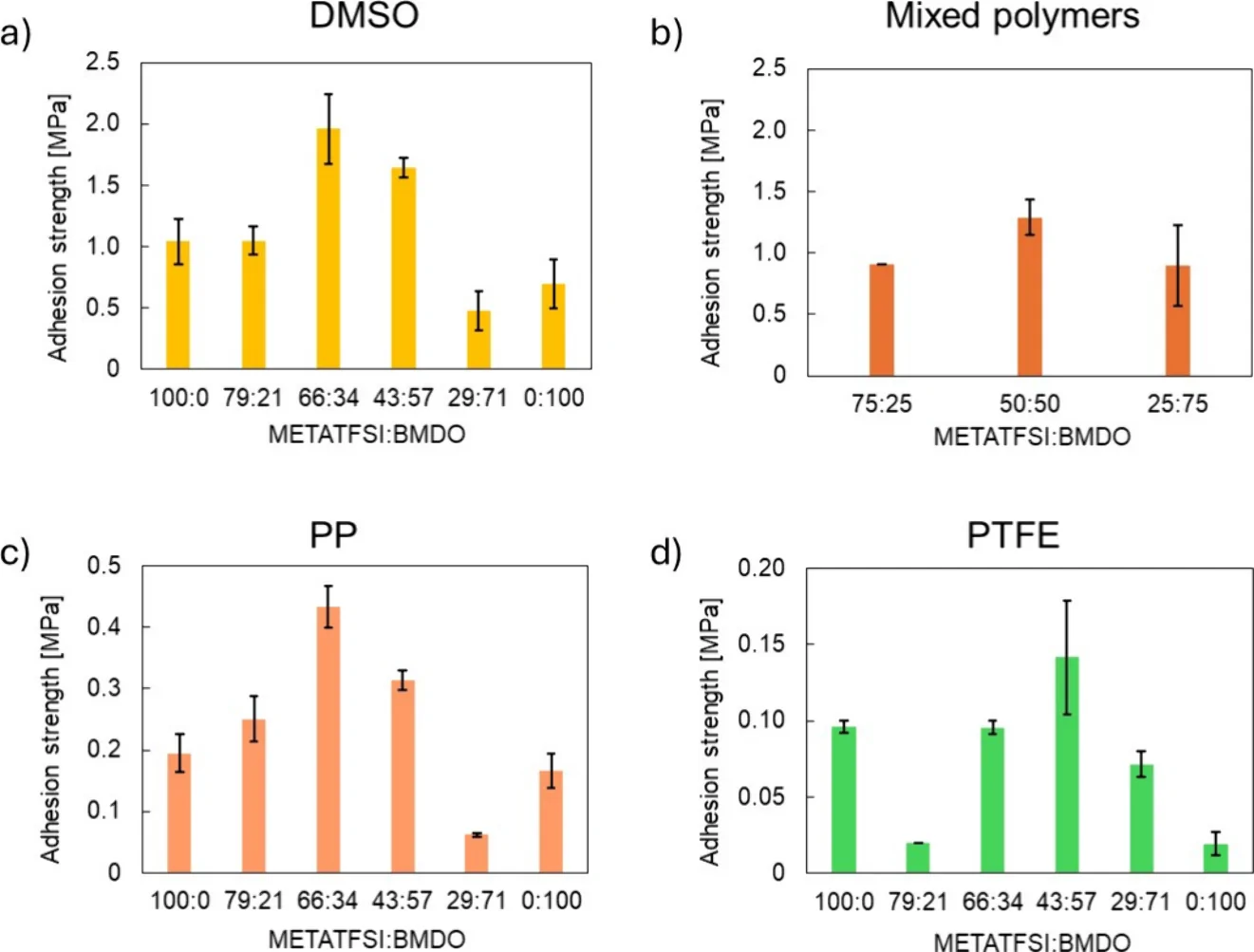
(a) Adhesive strengths of P­(METATFSI-*co*-BMDO) samples evaluated using DMSO as the solvent on Al plates. Adhesion experiments were performed at 60 °C to ensure complete solvent evaporation. (b) Adhesive strengths of mixed polymer systems comprising PMETATFSI and PBMDO homopolymers at various mixing ratios, with Al plates used to examine the contribution of intermolecular cation−π interactions. (c, d) Adhesive strengths of P­(METATFSI-*co*-BMDO) on (c) polypropylene (PP) and (d) polytetrafluoroethylene (PTFE) substrates as functions of copolymer composition, which demonstrates that effective adhesion to low-surface-energy materials is possible through a combination of cation−π-induced cohesion and interfacial ionic/dipolar interactions. The polymer concentration used for adhesion was 20 wt % in all experiments.

We also successfully bonded hydrophobic polypropylene (PP) substrates. PP is extensively used in numerous applications owing to its excellent chemical resistance and mechanical properties; however, it is regarded to be a difficult-to-adhere material, with reliable bonding generally difficult to achieve without modifying the surface via treatment with a primer.^34^ P­(METATFSI-*co*-BMDO) exhibited the same adhesive-strength trend toward PP as was observed for aluminum: maximum adhesive strength was observed for the copolymer with a 66:34 METATFSI/BMDO ratio, with lower strengths recorded for compositions with other ratios (Figure 5c).

The ability to adhere to fluoropolymeric substrates is particularly noteworthy, as such materials, including polytetrafluoroethylene (PTFE), are well-known to exhibit extremely low surface energies and poor adhesion characteristics.^35–37^ Even PTFE, as a representative material, exhibited a similar adhesion trend to those observed for PP and aluminum. P­(METATFSI-*co*-BMDO) exhibited maximum adhesive strength toward PTFE at a 43:57 METATFSI/BMDO ratio. In addition, the PMETATFSI homopolymer also exhibited a relatively high adhesive strength toward this fluoropolymer. This behavior is attributable to dipole–dipole and ion–dipole interactions between the trifluoromethyl groups or cationic moieties of the adhesive and the PTFE surface, which enhances interfacial adhesion. The decrease observed for the 79:21 copolymer may reflect a reduction in this fluorinated character before sufficient cohesive reinforcement through cation−π interactions is established. These results demonstrate that even PTFE, which has an extremely low surface energy and is generally difficult to bond, can be optimally bonded using an appropriate METATFSI/BMDO ratio by exploiting the combination of enhanced cohesive strength arising from cation−π interactions and strong interfacial adhesion ascribable to specific interfacial interactions.

## Conclusions

We used radical ring-opening copolymerization to synthesize a series of P­(METATFSI-*co*-BMDO) copolymers as a new class of cationic-side-chain-bearing aromatic polyester. Incorporating the METATFSI ionic-liquid monomer was found to markedly promote BMDO ring-opening to deliver high polymerization yields and controllable copolymer compositions. The resulting copolymers exhibited pronounced cation−π interactions, as evidenced by their low solubilities and superior cohesive properties, while the ester linkages introduced by BMDO endow the polymer with hydrolytic main-chain degradability. Adhesion experiments revealed that P­(METATFSI-*co*-BMDO) functions as a high-performance adhesive for a wide range of substrates, including aluminum and low-surface-energy polytetrafluoroethylene (PTFE); its high adhesive strength is ascribable to synergy between enhanced cohesion driven by cation−π interactions within the polymer matrix and strong interfacial adhesion arising from ionic and dipolar interactions with the substrate surface. Overall, this study establishes radical ring-opening copolymerization as a powerful strategy for endowing degradable polyester adhesives with strong noncovalent cation−π interactions. Accordingly, P­(METATFSI-*co*-BMDO) is a promising, sustainable adhesive platform that combines high adhesion performance with main-chain degradability and is applicable to challenging substrates that are typically difficult to bond.

## Supplementary Material



## Notes

^1^H NMR spectra of degradation products (PDF)
