# Supplementary material for: Adhesive Properties of Aromatic Polyesters with Cationic Side Chains Synthesized via Radical Ring-Opening Copolymerization
Source: ACS Omega. 2026 Aug 2;11(32):48634–41. doi: 10.1021/acsomega.6c06129 (PMC13491920; doi:10.1021/acsomega.6c06129)
Supplement: Supplementary file 1 [file ao6c06129_si_001.pdf]

Supporting Information

for

Adhesive Properties of Aromatic Polyesters with Cationic Side Chains Synthesized via Radical Ring-Opening Copolymerization

Gaku Sugimoto[1], Yoshiaki Kobayashi[1], Syuuhei Komatsu[1,2], Shohei Shiomoto[1,3], Akihiko Kikuchi[1,3], Taka-Aki Asoh[1]*

[1]Department of Materials Science and Technology, Tokyo University of Science

[2]Faculty of Pharmacy and Pharmaceutical Sciences, Josai University

[3]Department of Medical and Robotic Engineering Design, Tokyo University of Science

Degradation Test

A cast film was prepared by dissolving 0.5 g of P(METATFSI-*co*-BMDO) in 4.5 g of acetone, followed by casting the solution onto a Teflon dish and drying at 30 °C for 24 h. Film specimens (10–20 mg) cut from the cast film were immersed either in 1 mL of D_2O or in 1 mL of 0.1 mol L^{-1} NaOH in D_2O. DSS-d_6 standard solution was added to all samples as an internal reference. After incubation at 25 °C for two weeks, the supernatant solutions were collected and analyzed by ^1H NMR spectroscopy.

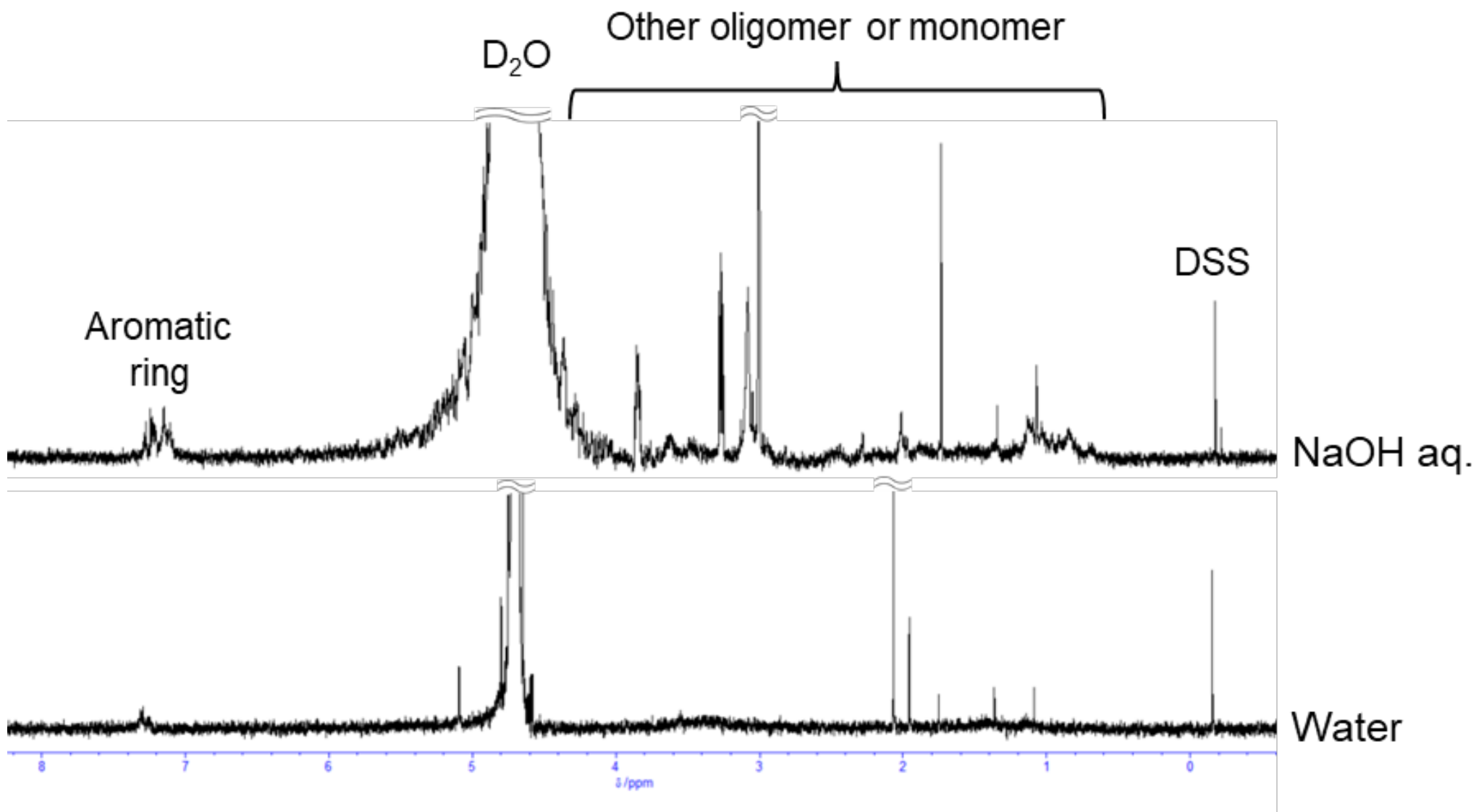

Figure S1 ^1H NMR spectra of the immersion solutions after incubation of P(METATFSI-*co*-BMDO) cast films in 0.1 mol L^{-1} NaOH in D_2O (top) and D_2O (bottom) and at 25 °C for two weeks. DSS-d_6 was used as an internal reference.
